# Impact of artifact removal on ChIP quality metrics in ChIP-seq and ChIP-exo data

**DOI:** 10.3389/fgene.2014.00075

**Published:** 2014-04-10

**Authors:** Thomas S. Carroll, Ziwei Liang, Rafik Salama, Rory Stark, Ines de Santiago

**Affiliations:** ^1^Cambridge Institute CRUK, University of CambridgeCambridge, UK; ^2^Lymphocyte Development, MRC Clinical Sciences Centre, Imperial CollegeLondon, UK

**Keywords:** ChIP-exo, ChIP-seq, QC, blacklist, duplicates

## Abstract

With the advent of ChIP-seq multiplexing technologies and the subsequent increase in ChIP-seq throughput, the development of working standards for the quality assessment of ChIP-seq studies has received significant attention. The ENCODE consortium's large scale analysis of transcription factor binding and epigenetic marks as well as concordant work on ChIP-seq by other laboratories has established a new generation of ChIP-seq quality control measures. The use of these metrics alongside common processing steps has however not been evaluated. In this study, we investigate the effects of blacklisting and removal of duplicated reads on established metrics of ChIP-seq quality and show that the interpretation of these metrics is highly dependent on the ChIP-seq preprocessing steps applied. Further to this we perform the first investigation of the use of these metrics for ChIP-exo data and make recommendations for the adaptation of the NSC statistic to allow for the assessment of ChIP-exo efficiency.

## Introduction

ChIP-seq couples chromatin immunoprecipitation with high throughput sequencing technologies to allow for the genome wide identification of transcription factor (TF) binding sites and epigenetic marks. The use of high throughput sequencing circumvents many of the limitations seen previously with ChIP-chip array based methods including probe specific biases and the physical limitations on the proportions of genomes which may be represented (Schmidt et al., 2008; Ho et al., 2011). ChIP-seq however inherits many of the technical artifacts found with ChIP enrichment analysis (non-specific binding of DNA, uneven fragmentation efficiency) as well as incurs novel problems associated to high-throughput sequencing (Park, 2009).

Following the papers first describing ChIP-seq (Barski et al., 2007; Johnson et al., 2007; Mikkelsen et al., 2007), the identification and removal of technical noise from ChIP-seq data has led to the development of common processing procedures (Kharchenko et al., 2008; Kidder et al., 2011; Bailey et al., 2013) and more recently the publication of standards for ChIP-seq quality control (Landt et al., 2012; Marinov et al., 2013). With the increase in sequencing output and the use of multiplexing technologies, such standards not only provide a more quantitative and unequivocal assessment of quality than can be established through visualization in a genome browser but also allow for the required high throughput classification of ChIP-seq quality.

In this study we investigate the application of such standards to classical ChIP-seq as well as ChIP-exo sequencing and evaluate the influence of common processing and filtering steps on these metrics. From the investigation of over 400 publically available ChIP-seq and ChIP-exo datasets, we identify the influence of common areas of aberrant signal on established ChIP quality metrics as well as highlight the importance of iterative quality assessment over ChIP-seq processing steps.

## Materials and methods

### Retrieval of sequencing data

TF and histone ChIP-seq was selected from the ENCODE/SYDH (The ENCODE Project Consortium, 2012) and CRUK datasets (310 and 145 datasets, respectively). For ChIP-exo data only TF data were included. Well characterized and replicated epigenetic factors and marks were selected for inclusion in this study and all data downloaded from the European Nucleotide Archive (ENA; http://www.ebi.ac.uk/ena/). Polymerase data was omitted from this study due to differential pattern of binding across transcriptional start sites and genes. ENA and SRA accession numbers for ENCODE/SYDH, CRUK ChIP-seq, and CRUK ChIP-exo datasets used in this study are included in the Supplementary Materials.

### Blacklisted regions

The DAC and DER blacklisted regions were downloaded from UCSC table browser (http://hgwdev.cse.ucsc.edu/cgi-bin/hgFileUi?db=hg19&g=wgEncodeMapability) (Fujita et al., 2011). The UHS regions were retrieved from (https://sites.google.com/site/anshulkundaje/projects/blacklists). Analysis of overlaps and read counts within blacklisted regions was performed using the GenomicRanges Bioconductor package version 1.8.13 with R 2.15.1 (Gentleman et al., 2004).

### Alignment and data processing

ChIP-seq and ChIP-exo reads were aligned to UCSC GRCh37 genome (February 2009 build) using BWA version 0.5.9 (Li and Durbin, 2010). For consistency, all reads were trimmed to a common length (28 bp, the smallest read length across all datasets). Reads were filtered to the male set of chromosomes omitting random contigs using Pysam 0.7.5. Calculation of read classes and proportions of blacklisted reads were performed using customs scripts implemented in Pysam 0.7.5 (http://code.google.com/p/pysam/).

### Calculation of quality metric and cross-correlation profiles

SSD metrics were calculated using the htSeqTools Bioconductor package for a representative chromosome (chromosome 1) (Gentleman et al., 2004; Planet et al., 2012). Cross-correlation profiles, RSC and NSC metrics for complete samples were performed using ccQualityControl version 1.1 (Marinov et al., 2013). Analysis of cross-correlation across DAC blacklists, peaks and read duplicates was performed using custom scripts and the GenomicRanges (version 1.8.13) and Rtracklayer (version 1.23.16) Bioconductor libraries (Gentleman et al., 2004) following previously described methodology (Kharchenko et al., 2008).

## Results

### Duplicate filtering and library complexity

High-throughput sequencing provides a measure of the frequency of sequence fragments from within the starting DNA fragment library and it is the variety of fragment sequences within this DNA pool that is defined as the library complexity (Landt et al., 2012). Sequence reads and read pairs mapping to the same position on the genome are termed duplicates and the frequency of occurrence of such duplicates is used as a metric of library complexity, with lower complexity libraries often being characterized by a higher rate of read duplication (Landt et al., 2012; Bailey et al., 2013).

The treatment of read duplicates and their use as a measure of library and sequencing quality varies between high-throughput sequencing applications. For whole genome and exome sequencing, the exclusion of duplicated reads is commonly performed to remove potential PCR amplification artifacts where PCR errors may be propagated leading to false positives in the identification of single nucleotide polymorphisms (Bainbridge et al., 2010). In contrast to this, in RNA-seq data, the measurements of quantitative changes in gene expression coupled with the expected large dynamic range of RNA molecules within a cell and between cell populations or tissues require a greater dynamic range of sequence depths than may be observed following the removal of duplicates.

The removal of duplicates from ChIP-seq data has been established as a common processing step in order to remove artifacts from PCR amplification bias and sources of aberrant signal (Zhang et al., 2008; Landt et al., 2012; Bailey et al., 2013). The proportion of duplicates within a data set alongside the total number of sequence reads has been used a measure of ChIP-quality and more recently formalized by the ENCODE consortium as the Non-Redundant Fraction (NRF) (Landt et al., 2012). Guidelines for NRF suggest that less than 20% of reads should be duplicates for 10 million reads sequenced (Landt et al., 2012).

Early ChIP-seq methodology suggested an expected duplication rate based on the number of sequence reads and the size of the mappable genome (Zhang et al., 2008; Zang et al., 2009). Although accounting for sequence depth, the use of all potential fragments from the mappable genome as the expected library complexity may overestimate the true complexity of libraries generated from epigenetic factors. An example can be taken from the ChIP-seq datasets here, where peaks from ER ChIP-exo (ERR336950) and ChIP-seq (ERR336952) can be seen to cover only ~ 0.17% and 0.24% of the genome, respectively. Analysis of duplication rates in ChIP-seq observed from single-end mapping using paired-end data has shown that the duplication rate for single end sequencing is overestimated and that this overestimation leads to ChIP-signal being preferentially lost within ChIP enriched regions (Chen et al., 2012). The exclusion of duplicated reads and read pairs from high through sequencing data limits the upper bounds of potential read depth on the genome and so restricts the observable dynamic range of ChIP signal. For single-end sequencing this cap on potential sequencing depth is the number of reads on either strand which may cover a genomic position uniquely and hence the potential range of signal is twice the read length.

Historically, ChIP-seq has been used to map potential binding events and epigenetic modifications without regard for quantification of the degree of signal observed within these events (Bailey et al., 2013). In this role and coupled with the limitations on signal height imposed from duplicate removal, the saturation of ChIP signal as a sacrifice for the discovery of less frequent epigenetic events may often occur.

### Blacklisting regions

In contrast to the genome wide removal of aberrant signal by filtering duplicated reads, specific genomic regions associated with artifact signal may be removed prior to further ChIP-seq analysis (Kharchenko et al., 2008; Bailey et al., 2013; Hoffman et al., 2013). The exclusion of these regions aims to remove sources of artifact signal caused by biases from chromatin accessibility and ambiguous alignment. The reduction of such noise prior to ChIP-seq analysis has been suggested to improve the estimation of fragment length and normalization of signal between samples and so increase accuracy of both peak calling and comparative ChIP analysis (Kharchenko et al., 2008; Bailey et al., 2013; Hoffman et al., 2013).

Kharchenko et al. first proposed the exclusion of reads from artifact regions by using signal generated from input samples (Kharchenko et al., 2008). In this study, three distinct classes of signal artifact for ChIP-seq were identified as (1) high signal and narrow enrichment, (2) structured narrow enrichment indistinguishable from ChIP enrichment and (3) long (> 1000 bp), unstructured regions of signal (Kharchenko et al., 2008). The effects of artifactual high signal regions on the identification of putative binding sites are in the most part eliminated through the use of appropriate input controls. However, regardless of the use of an input, many properties of a sample important to further ChIP-seq analysis remain confounded by sources of high artifact signal (Kharchenko et al., 2008).

Recent work by Furey and Kudaje as part of the ENCODE project has led to the creation of two sets of “blacklist” regions for the human genome which are believed to contain experiment and cell-type independent areas of high artifact signals (Hoffman et al., 2013; Kundaje, 2013). These regions therefore potentially provide a methodology for the removal of artifact signal common to all human ChIP-seq analysis.

**Figure 1:**
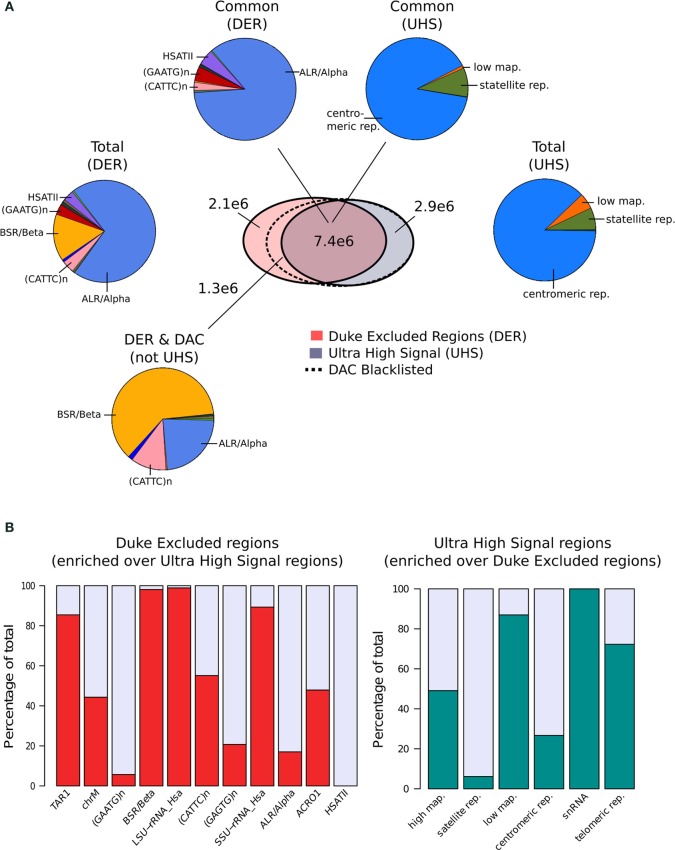
**(A)** The venn-diagram represents the genomic overlap between DAC consensus, UHS, and DER blacklists. Pie charts show the proportions of blacklist classes contained within overlapping and unique regions of the DAC consensus, UHS, and DER blacklists. **(B)** Bar charts show the relative enrichment of blacklist classes unique to either DER and UHS blacklist regions.

Terry Furey at Duke University (Kundaje, 2013) created the set of Duke Excluded Regions (DER). Although its construction is not fully described, this blacklist consists of 11 distinct repeat classes making up 1648 regions covering ~0.34% (~10Mb) of the human genome. Of the repeat classes included, around 85% of the DER is constructed of just two repeat classes with ALR/Alpha and BSR/Beta repeats representing 70 and 15%, respectively, of the total (Figure 1A).

In parallel, Anshul Kudaje produced the Ultra High Signal Artifact (UHS) regions derived from a subset of the ENCODE open chromatin and input sequence data (Hoffman et al., 2013; Kundaje, 2013). This represents a set of manually curated genomic regions found to contain high degrees of artifact signal. By identifying regions showing extreme depths in open chromatin and input control for uniquely mapped reads and combining this with measures of mappability, an initial set of ultra-high signal regions was identified (Kundaje, 2013). With manual curation of these regions alongside annotation of repeat class and gene locations, the final UHS region set was created (Kundaje, 2013). The UHS regions contain 226 genomic locations but despite the inclusion of less distinct regions than the DER set covers a similar genomic proportion of 0.33%. As with the DER, the UHS set consists of six classes of regions with 88% of all regions comprised of centromeric repeats (Figure 1A).

These two separately derived blacklisted regions have considerable overlap with ~30% of DER and 67% of UHS regions shared between the two sets. This overlap is even greater when considered as genome covered with 68% percent of UHS's and 71% percent of the DER's genomic coverage in common (Figure 1A). Analysis of the regions common and exclusive to each set shows that BSR/Beta, Tar1 and rRNA repeats are overrepresented in the DER only sets (Figure 1B). In keeping with the derivation of the DER from repeat classes, 50% of high mappability islands are found only within the UHS's regions (93% when excluding ChrM), whereas large proportions of the centromeric and satellite repeats, analogous to DER's Alpha and Beta repeats, are common to both sets (Figure 1B).

Following their creation and characterization, the DER and the UHS blacklists were amalgamated into the DAC Consensus Excluded Regions (Kundaje, 2013). Assessment of the regions unique to the DER identified a further 38% of DER regions not included within UHR regions as having “medium scale signal” whereas the remaining repeat regions were found to be low signal (Kundaje, 2013). These regions are enriched for both CATTC and BSR/Beta repeats and their supplement to the UHS regions produced the final DAC Consensus Excluded Region blacklist commonly used for exclusion of artifact signal.

**Figure 2:**
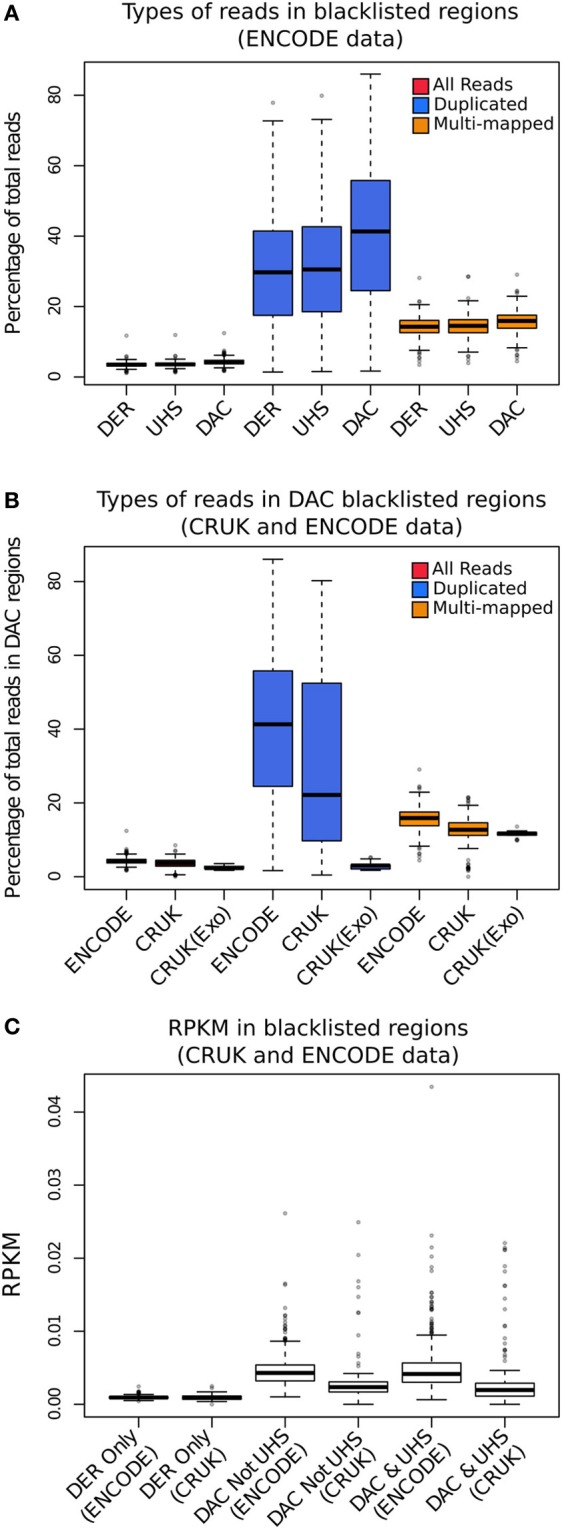
**(A)** The boxplots show the percentage of total reads for all (red), duplicated (blue), and multi-mapped reads (orange) within the DAC consensus, UHS, and DER blacklists for ENCODE/SYDH datasets. **(B)** The boxplots show the percentage of total reads for all (red), duplicated (blue), and multi-mapped reads (orange) within the DAC consensus for ENCODE/SYDH and CRUK datasets. **(C)** Boxplots illustrating the range of RPKM within blacklist classes for DER only, DAC consensus not within UHS and the overlapping DAC consensus and UHS regions.

In order to investigate the proportion of signal attributable to such blacklists, reads from different read classes (all, duplicated and multi-mapped reads) were counted within all blacklists' regions for the ENCODE, CRUK, and ChIP-exo data sets. All data sets showed an enrichment of reads within blacklisted regions (Figure 2A), with ~10-fold for ENCODE and CRUK ChIP-seq datasets and ~5-fold for ChIP-exo sets (Figure 2B) highlighting their strong acquisition of background signal. The number of reads mapping to the DER, the UHS, and the DAC blacklisted regions demonstrates how the DER-only regions (DER regions not amalgamated in the DAC consensus set) are largely devoid of signal when compared to DAC consensus regions (Figure 2C) and so their exclusion from the list of blacklisted regions has little effect on the removal of artifact signal across all sets.

Across all datasets and blacklists there is enrichment for reads mapping to more than one location in keeping with repeat classes constituting large portions of the blacklists (orange boxplots, Figure 2B). An even greater enrichment is seen in ChIP-seq samples for duplicated reads within blacklisted regions and the proportion of total reads as duplicates and the degree of signal within blacklist regions can be found to be highly correlated (blue boxplots, Figure 2B). The enrichment of duplicated reads within blacklists is unsurprising given the common association with artifact signal but this observation exemplifies the classification of duplicates contributing to aberrant signal from those within areas of genuine ChIP enrichment. In contrast to this, ChIP-exo samples can be found to have consistently lower proportions of duplicates in blacklisted regions (blue boxplots, Figure 2B). This finding may reflect either the lower presence of artifact signal or an increased rate of duplication previously observed within ChIP-exo peaks (Serandour et al., 2013).

### Inequality of coverage

ChIP-seq data is most often visually or statistically interrogated to identify points or stretches of signal enriched above expected by the use of background signal such as that of an input control. Assessment of the global extent of signal depth allows for the quantification of enrichment for signal within a ChIP sample or the degree of aberrant signal within an input control. Calculation and visualization of the number of base pairs at varying depths of signal allow for a qualitative inspection of enrichment but more recently two metrics describing the inequality of coverage across ChIP-seq datasets have been described (Planet et al., 2012).

Standardized Standard Deviation (SSD) is calculated from the weighted mean of the standard deviation of the depth of coverage across chromosomes normalized to the total number of reads sequenced (Planet et al., 2012). Since SSD describes the variation in signal depth across the genome it is sensitive to regions of high signal such as that observed in blacklisted regions for both input and ChIP samples.

**Figure 3:**
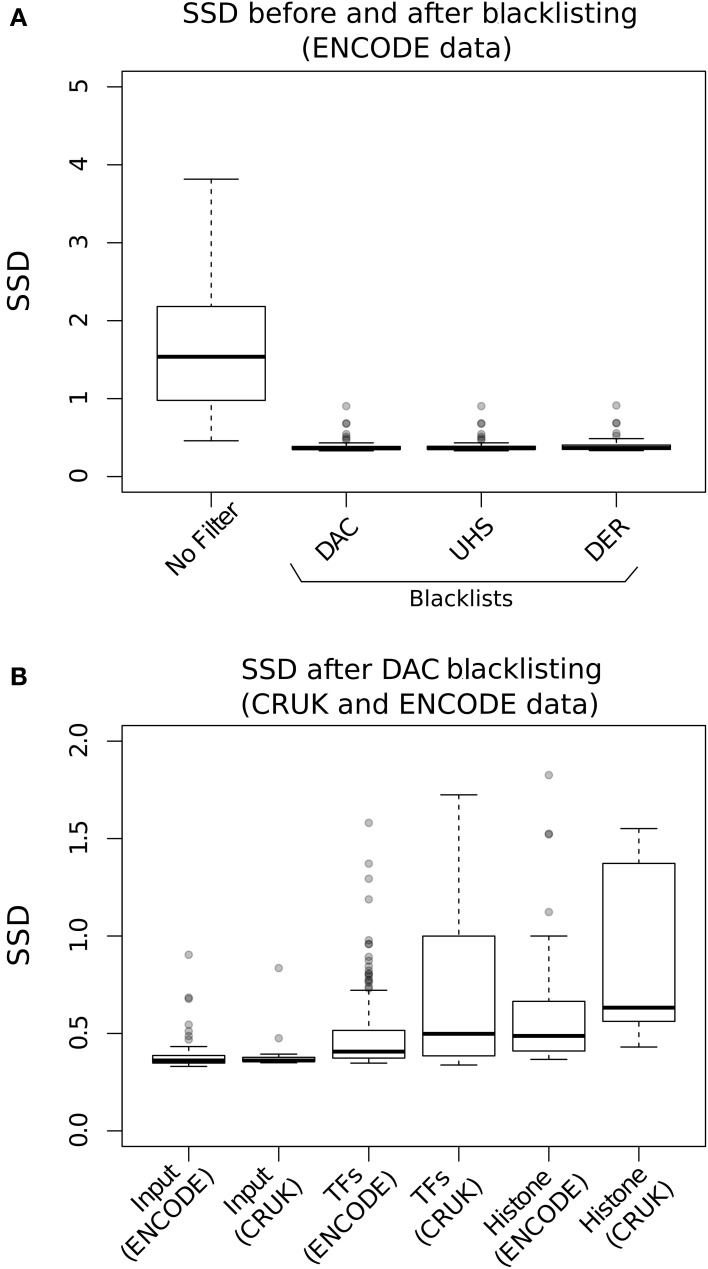
**(A)** The Boxplots show the range of SDD values for CRUK and ENCODE/SYDH input samples with no filtering steps applied and after filtering of signal from DAC consensus, UHS, and DER blacklists. **(B)** Boxplots of the SSD scores for input, transcription factors (TFs) and histone marks from ENCODE and CRUK datasets following blacklisting by the DAC consensus regions.

Evaluation of the effects of blacklisting on SDD identifies the highly significant reduction in both input and ChIP samples after blacklist removal and highlights the dominant effects of artifact signal on the calculation of SSD scores (Figure 3A). Blacklisting differentially reduces the mean and range of SSD scores for input compared to ChIP samples (Figure 3B) and so illustrates the effectiveness of blacklisting in removing the majority of regions that show artificially high signal while maintaining ChIP signal. After blacklisting, one should expect a reduced SSD score in the input when compared to the ChIP sample, and therefore the observation of a similar SSD score between both sample and input may act as a flag to further remove artifact regions from the genome.

### Estimations of fragment length

**Figure 4:**
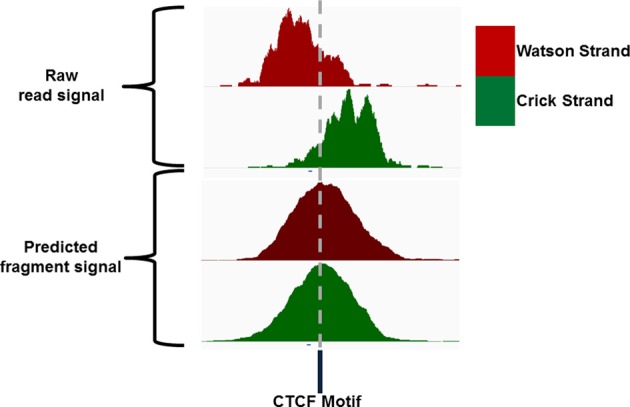
**IGV screenshot of an example CTCF ChIP signal showing the distribution of Watson and Crick signal around the CTCF motif and the distribution of Watson and Crick signal following extension of reads to the expected fragment length**.

Sequence reads generated from high throughput sequencing technologies typically only represent the 5′ and 3′ end portions of DNA fragments within the library pool. In ChIP-seq the reconstruction of the true fragments from the available sequence reads allow for a more accurate representation of ChIP-signal across the genome and a higher resolution of epigenetic marks and DNA binding sites (Figure 4) (Kharchenko et al., 2008). The requirements to identify potential splicing and genome structural rearrangement events by RNA-seq and DNA resequencing have led to the more frequent use of paired end sequencing within these technologies but the additional time and financial costs often prohibit their use for ChIP-seq. The inference of fragment length from single end ChIP-seq has therefore received much attention and many bioinformatic methods have been described for its prediction (Kharchenko et al., 2008; Zhang et al., 2008; Sarkar et al., 2009; Ramachandran et al., 2013).

Single end sequencing of these ChIP DNA fragments leads to the structured arrangement of clusters of reads from the Watson and Crick strands separated around the true point of maximal ChIP enrichment (Figure 4) (Kharchenko et al., 2008). It is the assessment of distances between these two distributions around the central expected binding events that is employed by many methods of fragment length prediction (Kharchenko et al., 2008; Zhang et al., 2008; Sarkar et al., 2009; Ramachandran et al., 2013).

The peak calling algorithm MACS performs fragment length estimation as an initial step in its peak calling procedure (Zhang et al., 2008). Proximal peaks on the Watson and Crick stand showing enrichment above background between a set range are defined as “paired peaks” (Zhang et al., 2008). By measuring the distance between these paired peaks an estimation of the fragment length may be made (Zhang et al., 2008). This method relies on the initial identification of paired peaks and so fails to predict fragment length should the criteria for paired peaks not be satisfied and is sensitive to artifact signal meeting paired peak criteria.

**Figure 5:**
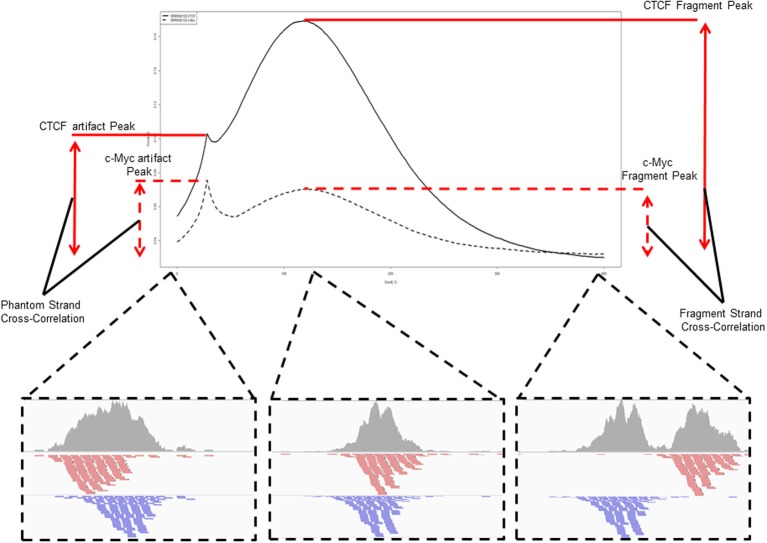
**An example and illustration of the assessment of cross-correlation following shifting of the reads on the Watson strand.** The cross-correlation of the CTCF ChIP sample (SRR568129) shows the dominance of the fragment-length cross correlation peak over the read-length cross correlation peak. The c-Myc ChIP sample (SRR568130) in contrast shows greater cross-correlation at the read-length peak than at the expected fragment length highlighting potential problems in fragment length prediction for that sample.

A popular method for predicting fragment length is the method of cross-correlation analysis (Kharchenko et al., 2008). In this method the correlation between signal of the 5′ end of reads on the Watson and Crick strands is assessed after successive shifts of the reads on the Watson strand and the point of maximum correlation between the two strands is used as an estimation of fragment length (Figure 5) (Kharchenko et al., 2008).

**Figure 6:**
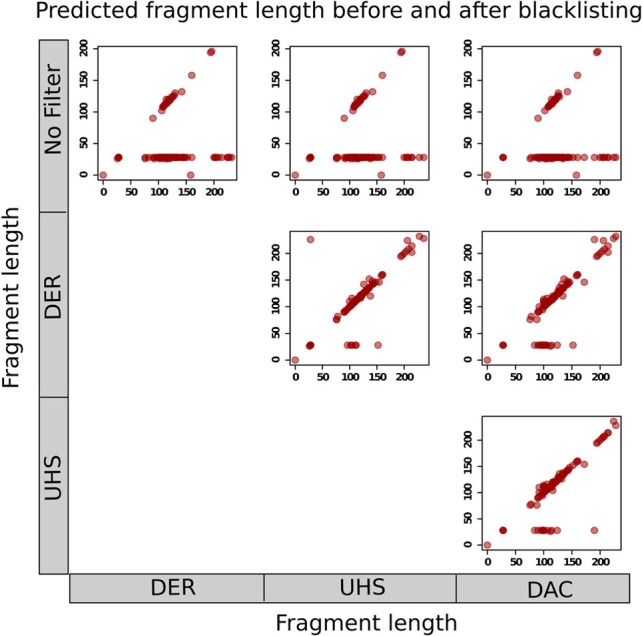
**Scatterplots show the fragment lengths predicted by cross-correlation analysis for transcription factor datasets from the ENCODE/SYDH set, with no filtering and following blacklisting by the DAC consensus, UHS, and DER blacklists**.

The effects of blacklisting on fragment length estimation of ChIP samples by these methods can be seen to be dramatic (Figure 6). Both the cross-correlation and MACS method can be seen to be positively influenced by the removal of aberrant signal from blacklisted regions and by duplicate filtering. When no filtering steps are applied, the fragment length is predicted to be the same as the read length in many of the ChIP samples whereas the prediction of sensible fragment lengths is rescued following blacklisting and removal of duplicated reads (Figure 6).

In fact, highly duplicated genomic positions result in distributions of Watson and Crick 5' read ends separated by the read length around the center of the duplicated read stack. This phenomenon introduces a spike in cross-correlation at the read length (read-length peak) which may supersede that of the fragment length when assessing shift with maximum correlation for ChIP-seq as well as result in paired peaks on the Watson and Crick stand separated by the read length, thus leading to the incorrect prediction of the fragment length as the read length.

### Cross-correlation analysis and identification of artifact and ChIP signal

The use of cross-correlation to predict fragment length provides further information about the overall quality of a ChIP-sample (Landt et al., 2012; Marinov et al., 2013). By assessing the correlation at the fragment length and at the read length, an evaluation of the degree of ChIP and artifact signal within a sample may be made. Metrics to quantify the fragment length signal and the ratio of fragment length signal to read length signal have been coined as the Normalized Cross Correlation (NSC) and Relative Cross Correlation (RSC) metrics (Landt et al., 2012; Marinov et al., 2013).

In contrast to SSD, which is agnostic of signal structure, RSC and NSC metrics are dependent on the clustering of Watson and Crick strand reads around binding sites. Since for TFs, fragment lengths are often greater than the size of the DNA binding event, the distinct clustering of Watson and Crick reads around this site is very apparent whereas for longer epigenetic marks this clustering may be more diffuse. This highlights an important distinction between SSD and NSC/RSC metrics where ChIP samples with broad signal enrichment (e.g., histone marks) typically achieve higher SSD and lower NSC or RSC scores than those with sharper signal enrichment over narrow regions (e.g., TFs).

**Figure 7:**
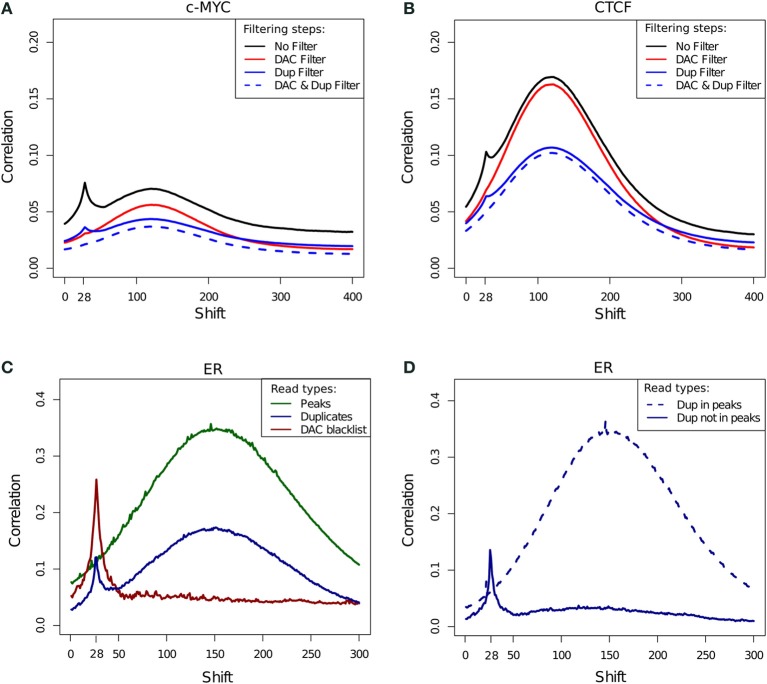
**(A,B)** Example cross-correlation profiles for a c-Myc **(A)** and a CTCF **(B)** sample (SRR568130 and SRR568129, respectively). Cross-correlation profiles after no filtering, filtering of duplicated reads, exclusion of DAC consensus blacklist and simultaneous blacklisting and duplicate removal. **(C)** Cross correlation profiles of reads in DAC blacklisted regions, reads in peaks and duplicated reads for an example ER ChIP-seq sample (ERR336952). **(D)** Cross correlation profiles for duplicated reads inside and outside of peaks for an example ER ChIP-seq sample (ERR336952).

The cross-correlation profile for c-Myc (SRR568130; Figure 7A) and CTCF (SRR568129; Figure 7B) ChIP-seq samples exemplifies the contribution of the DAC blacklist and duplicates to cross-correlation profiles. After blacklisting the total loss of the read-length peak at the 28 bp position is observed, while duplicate removal confers a more subtle effect (Figures 7A,B).

In order to investigate the effects of differing filtering steps on cross-correlation profiles and hence NSC/RSC scores, reads were separated into those overlapping peaks, overlapping blacklists and duplicated reads. Whereas cross-correlation profiles derived from reads in peaks show the expected hump around the fragment length, the cross-correlation profile obtained by considering solely the DAC blacklist shows only the read-length spike illustrating the presence of the artifact signal within blacklisted regions and its influence in the read-length cross-correlation spikes for ChIP and input samples (Figure 7C). Interestingly the cross-correlation of duplicated reads shows peaks at both the read length and the fragment length, which is indicative of both artifact and structured ChIP signal within the duplicated reads. Further sub setting of duplicated reads to those within and outside of peaks confirms the presence of two classes of duplicated signal with those outside of peaks contributing to artifact signal and those within peaks contributing to structured ChIP signal over binding sites (Figure 7D).

To systematically evaluate the effect of filtering steps on ChIP and background signal, cross-correlation profiles and their fragment-length and relative strand cross-correlation scores were assessed after blacklisting, duplicate removal and both simultaneously.

**Figure 8:**
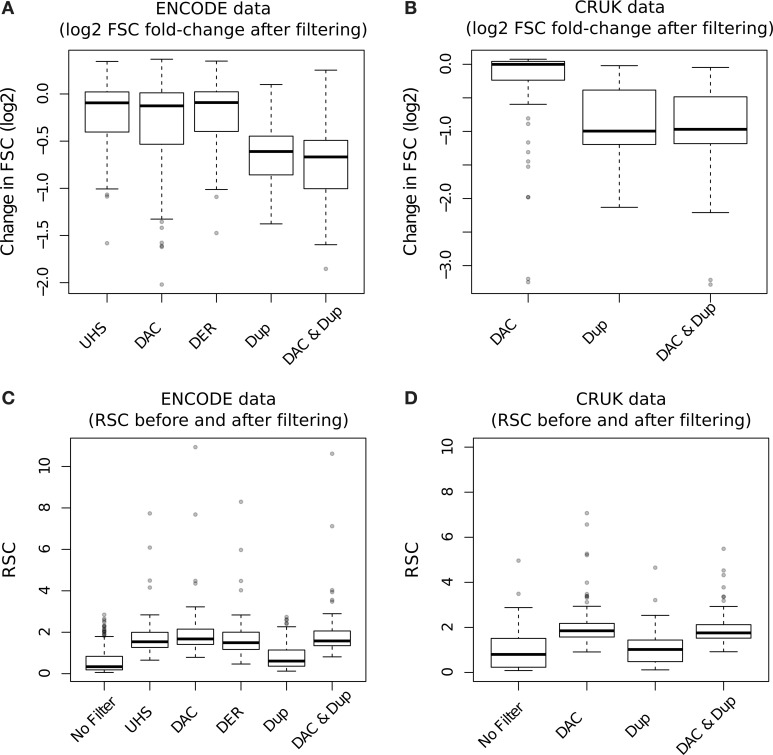
**(A,B)** Boxplots of the RSC scores for TF datasets from the ENCODE/SYDH (A) and from CRUK (B) sets after differing filtering steps. For the CRUK set only the DAC consensus set was used to evaluate the effect of blacklisting given its observed greater enrichment for artifact signal over the DER/UHS sets within ENCODE data. **(C,D)** Boxplots of the change in cross-correlation signal at the fragment length (fragment strand cross-correlation; FSC) for TF datasets from the ENCODE/SYDH **(C)** and from CRUK **(D)** sets following the removal of blacklisted regions, duplicated reads and removal of both blacklisted regions and duplicated reads.

The effect of blacklisting and/or duplicate removal on FSC scores is shown in Figure 8A (ENCODE samples) and Figure 8B (TF/CRUK samples) by the ratios of FSC scores after filtering to that observed with no filtering. Duplication filtering is seen to have more influence on the FSC than any blacklist filtering illustrating the greater depletion of fragment length signal by duplicate removal and hence depletion of signal related to binding events.

In keeping with the observations of DAC blacklisted reads contributing to the read-length cross-correlation peak, an increase in RSC scores across both ENCODE (Figure 8C) and CRUK (TFs only; Figure 8D) datasets was observed following removal of DAC regions. Duplication filtering can be seen to have no significant effect on RSC due to its expected reduction of both fragment-length and read-length cross-correlation peaks. Further to this, the additional step of duplication filtering has little effect on RSC after blacklisting but shows a drop in the fragment-length cross-correlation peak as seen with standard duplication filtering (Figures 8C,D). The lack of change in RSC observed here is due to prior blacklisting which removes reads contributing to the read-length cross-correlation peak and so the read length score reflects a tail of fragment-length cross-correlation peak as opposed to true read-length peak. This highlights an important caveat of RSC scores where removal of aberrant signal may cause RSC to reflect the width of fragment-length cross-correlation peak instead of the extent of background signal.

### Application of cross-correlation analysis to ChIP-exo data

**Figure 9:**
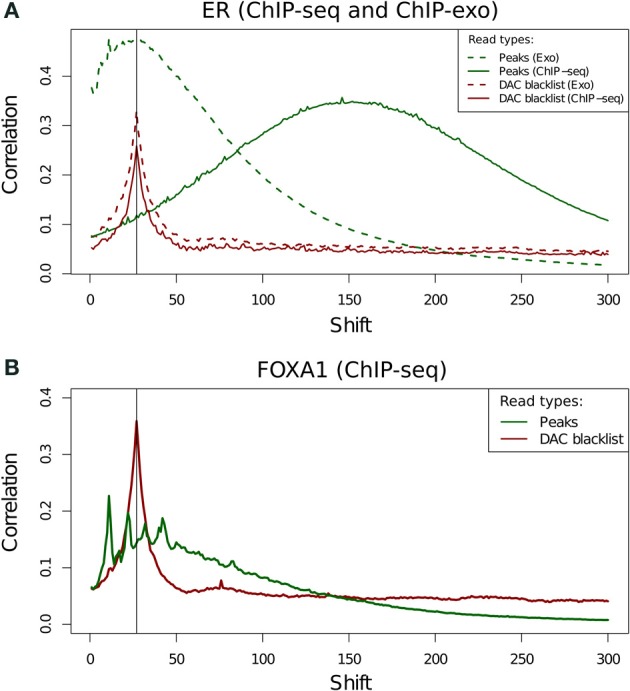
**(A,B)** Cross-correlation profiles for reads in peaks and reads in DAC consensus blacklist for ChIP-seq and ChIP-exo ER ChIP **(A)** and for ChIP-exo FoxA1 ChIP **(B)**.

The use of cross-correlation analysis and NSC/RSC metrics for ChIP-exo data has not been previously investigated. Due to the enzymatic digestion of DNA fragments around binding sites cross-correlation profiles are expected to have a very different shape to that of successful ChIP. Neither the ER (Figure 9A) or FoxA1 ChIP-exo (Figure 9B) have any evidence of a conventional fragment-length peak and all spikes in cross correlation can be seen to be close to the read length. Separation of ChIP-exo reads into those overlapping peaks and blacklist regions, allows for the identification of the true profile of ChIP-exo enrichment and illustrates the persistent presence of the cross-correlation read length peak. This highlights the continued need of blacklisting to filter regions of artifact signal in this new technology despite their relatively lower rate of blacklisted signal (Figure 2B).

Cross-correlation profiles for ChIP-exo can be seen to be distinct between ER and FoxA1 ChIP. ER shows a broad enrichment over the read-length cross-correlation peak (Figure 9A, dashed lines) whereas FoxA1 (Figure 9B) shows a more complex profile of peaks around this point typically seen at the fragment length for ChIP-seq enzymatic fragmentation (Marinov et al., 2013). The co-occurrence of the artifact signal and ChIP signal cross-correlation peaks makes the disentanglement of different types of signal difficult and the calculation of NSC and RSC impossible using the standard methods.

**Figure 10:**
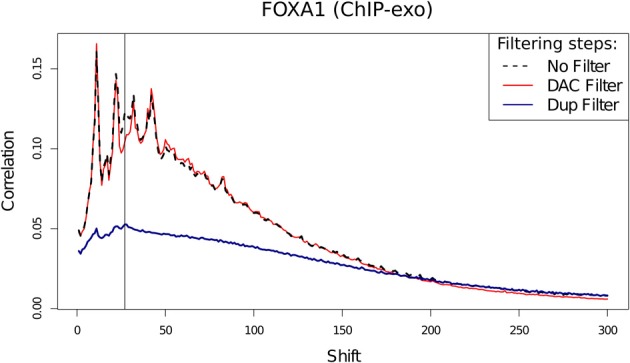
**Cross-correlation profile for FoxA1 ChIP-exo after no filtering, removal of DAC blacklisted regions and removal of duplicated reads**.

Assessment of the effects of filtering on ChIP-exo shows that duplicate filtering has a dramatic effect on the overall cross-correlation profile whereas blacklisting has a specific effect in FoxA1 at the 28 bp cross-correlation peak and little effect at the 12 bp peak (Figure 10). In this case, by identifying the elements of the cross-correlation profile relating to aberrant and ChIP signal, new metrics related to NSC and RSC may be calculated.

Following the removal of aberrant signal and the contribution of this to the read-length cross-correlation peak, the ratio between the highest and minimum values of cross-correlation may replace the use of typical NSC scoring for ChIP-exo quality and so provide an equivalent measure of ChIP efficiency. The use of a metric equivalent to RSC's evaluation of signal to noise in ChIP-seq however is confounded by the overlap between read-length and fragment-length cross-correlation peaks. Nonetheless, in ChIP-exo data the observation of loss of a defined read-length cross-correlation peak after removal of artifact signal can act as an indication of successful removal of artifact signal.

## Conclusions

The processing of ChIP-seq data and the evaluation of ChIP quality remains an area of continued research. Following recent publications of ChIP quality metrics and analysis standards, we have performed the first systematic evaluation of the effects of ChIP-seq pre-processing steps on such metrics and an assessment of their application to the emerging technology of ChIP-exo sequencing.

### Successive assessment of ChIP metrics over processing steps is required to capture ChIP-seq quality

The assessment of ChIP-quality by the visualization of ChIP signal within genome browsers can be subjective to the investigator and is prohibitive of large scale evaluation of quality. The use of metrics of ChIP-quality therefore provides more objective methods to evaluate ChIP success as well as allows for high throughput classification of ChIP data. These metrics are however dependent on processing and filtering steps applied and therefore their interpretation must be made in their context.

The removal of artifact signal can improve fragment length estimation and between sample normalization (Kharchenko et al., 2008; Bailey et al., 2013; Hoffman et al., 2013). The DAC blacklist regions provide a set of known artifact regions where enrichment for background signal has been found to be conserved across several human cell lines (Hoffman et al., 2013; Kundaje, 2013). The DAC blacklist is enriched for duplicated reads, has high variation in signal depths and directly contributes to artifact peak found within cross-correlation profiles. The presence of this peak can confound fragment length estimation and is a key component of the calculation of the RSC metric. The assessment of quality therefore is strongly influenced by the removal of these regions.

The SSD metric of signal inequality is highly sensitive to high signal artifact regions and so to evaluate ChIP enrichment masking of such regions is required prior to assessment of SSD. Furthermore, due its sensitivity to artifact regions, the SSD metric can be used as a flag for the persistence of artifact regions in input samples where higher scores for the input sample when compared to the ChIPed sample highlights the requirements for further artifact removal.

The RSC metric provides a measure of ChIP to artifact signal, however the removal of blacklisted regions has been shown to eliminate the presence of the artifact peak and so the interpretation of RSC after blacklisting is obscured. In contrast to SSD, the assessment of RSC should be performed prior to blacklisting and the inspection of cross-correlation profiles be made after blacklisting to confirm the loss of the read-length peak within the cross-correlation profile.

The treatment of duplicated reads in ChIP-seq varies between applications and the inclusion of duplicated reads is often performed in the context of differential affinity analysis (Ross-Innes et al., 2012; Bailey et al., 2013). Duplicated reads can be seen to contribute to both artifact and ChIP signal and the removal of duplicated reads significantly reduces ChIP signal across samples. The evaluation of ChIP quality following duplicate removal may therefore underestimate the extent of ChIP enrichment relative to background and so careful consideration of the contribution of duplicates to artifact regions and ChIP signal must be made prior to evaluation of NSC and RSC metrics.

From these finding, we show the importance of the iterative assessment of quality over the masking of blacklisted regions and removal of duplicated reads. We recommend the assessment of RSC and NSC prior to blacklisting or duplicate removal and SSD before and after these steps to capture the extent and success of blacklisting.

### ChIP-exo requires different processing and an adaptation of cross-correlation metrics

ChIP-exo sequencing presents a new methodology for genome wide ChIP analysis and provides a higher resolution and greater efficiency than seen for conventional ChIP (Serandour et al., 2013). An evaluation of the effects of common processing steps as well as the use of standard ChIP metrics has however not been previously performed.

The presence of artifact signal from blacklisted regions may be seen in ChIP-exo data but the degree of blacklisted signal was found to be consistently lower for this technology (Figure 2B). The significant loss of ChIP-related signal within cross-correlation analysis following duplication removal illustrates the greater contribution of duplicates to ChIP-exo enrichment signal. The removal of blacklists but the retention of duplicates can therefore be recommended for ChIP-exo processing.

The use of standard cross-correlation analysis in the evaluation of ChIP-exo quality is confounded by the co-occurrence of both the read-length and the fragment-length cross-correlation peaks. Although this prohibits the use of the RSC metric, by identifying the expected cross-correlation profile of enriched regions, an adapted NSC metric may be generated as the extent of maximum cross-correlation within this profile over the background cross-correlation following the blacklisting of aberrant signal.

### Conflict of interest statement

The authors declare that the research was conducted in the absence of any commercial or financial relationships that could be construed as a potential conflict of interest.
