# Supplementary material for: Impact of artifact removal on ChIP quality metrics in ChIP-seq and ChIP-exo data
Source: Front Genet. 2014 Apr 10;5:75. doi: 10.3389/fgene.2014.00075 (PMC3989762; doi:10.3389/fgene.2014.00075)
Supplement: Supplementary file 1 [file DataSheet1.DOCX]

Supplementary information

ENA and SRA accession numbers used in this study

**The ENA and SRA accession numbers for CRUK chip-seq and ChIP-exo datasets:**

ERP003828, SRP014854, SRP015695 and SRP032421.

**The SRA accession numbers for SYDH Encode datasets:**

SRR568131, SRR568132, SRR568129, SRR568130, SRR502663, SRR502664, SRR502620, SRR502621, SRR502288, SRR502289, SRR502686, SRR502687, SRR502448, SRR502449, SRR502450, SRR502451, SRR502452, SRR502628, SRR502629, SRR502269, SRR502270, SRR502168, SRR502169, SRR502215, SRR502286, SRR502287, SRR502531, SRR502532, SRR568149, SRR568150, SRR502341, SRR502342, SRR502343, SRR502344, SRR501969, SRR501970, SRR502521, SRR502522, SRR502527, SRR502528, SRR502630, SRR502631, SRR502162, SRR502163, SRR502292, SRR502293, SRR502445, SRR502446, SRR502447, SRR501949, SRR501950, SRR502290, SRR502291, SRR501946, SRR501947, SRR501948, SRR501944, SRR501945, SRR502280, SRR502281, SRR502454, SRR502455, SRR502456, SRR502457, SRR502458, SRR502278, SRR502279, SRR502437, SRR502438, SRR501937, SRR501938, SRR501939, SRR501940, SRR501941, SRR502284, SRR502285, SRR502442, SRR502443, SRR502444, SRR501934, SRR501935, SRR501936, SRR502282, SRR502283, SRR502453, SRR502463, SRR502464, SRR502465, SRR502466, SRR502028, SRR502029, SRR502296, SRR502297, SRR501916, SRR501917, SRR501918, SRR502033, SRR502034, SRR502294, SRR502295, SRR501929, SRR501930, SRR501931, SRR502406, SRR502407, SRR502090, SRR502145, SRR501965, SRR501966, SRR353507, SRR502469, SRR502576, SRR502041, SRR502025, SRR502026, SRR502355, SRR502356, SRR353505, SRR353665, SRR502063, SRR502099, SRR502473, SRR502474, SRR502475, SRR502476, SRR502477, SRR502478, SRR502479, SRR502480, SRR502481, SRR501960, SRR501961, SRR502388, SRR502389, SRR502103, SRR502104, SRR502642, SRR502643, SRR353506, SRR502008, SRR502009, SRR502010, SRR502271, SRR502272, SRR502273, SRR502274, SRR502419, SRR502420, SRR502611, SRR501993, SRR501994, SRR501991, SRR501992, SRR502626, SRR502627, SRR502494, SRR502495, SRR502077, SRR502408, SRR502409, SRR502198, SRR502199, SRR502413, SRR502414, SRR502196, SRR502197, SRR502211, SRR502212, SRR502238, SRR502239, SRR502317, SRR502318, SRR502421, SRR502422, SRR502500, SRR502501, SRR502602, SRR502603, SRR502204, SRR502205, SRR502109, SRR502110, SRR502225, SRR502226, SRR502228, SRR502237, SRR502606, SRR502641, SRR502667, SRR502668, SRR502429, SRR502430, SRR502003, SRR502004, SRR502192, SRR502193, SRR502376, SRR502377, SRR502327, SRR502328, SRR502329, SRR502330, SRR502361, SRR502362, SRR502363, SRR502364, SRR502267, SRR502268, SRR502370, SRR502371, SRR502224, SRR502504, SRR502612, SRR502613, SRR502614, SRR502615, SRR502616, SRR502617, SRR502691, SRR361464, SRR361465, SRR502275, SRR502276, SRR502277, SRR502357, SRR502358, SRR502694, SRR502695, SRR502102, SRR502227, SRR502640, SRR502589, SRR502054, SRR502306, SRR502538, SRR502539, SRR502027, SRR568184, SRR568185, SRR568166, SRR568167, SRR568170, SRR568171, SRR501957.
